# Integrating evolutionary and compositional features with ML and DL for robust and interpretable druggable protein prediction

**DOI:** 10.1007/s10822-026-00818-1

**Published:** 2026-06-30

**Authors:** Mujeebu Rehman, Qinghua Liu, Muhammad Javed, Ali Ghulam, Teerath Kumar

**Affiliations:** 1 School of Information and Communication, Guilin University of Electronic Technology, Guilin, 541004 China; 2 College of Mechanical Engineering, Donghua University, Shanghai, China; 3 Information Technology Centre, Sindh Agriculture University, Tandojam, Sindh Pakistan; 4 Department of Computing, Atlantic Technological University, Letterkenny, Ireland

**Keywords:** Computational drug discovery, Deep learning models, Machine learning models, Druggable proteins, Hybrid feature representation, Position-specific scoring matrix (PSSM), Dipeptide composition (DPC), Protein sequence analysis

## Abstract

Druggable proteins are essential targets in contemporary therapeutic discovery, and their precise identification is necessary for the progression of rational drug design. Traditional biochemical screening techniques are costly, laborious, and protracted, whereas current computational models typically attain only moderate efficacy and seldom offer robust statistical validation or biological interpretability. To mitigate these limitations, we offer a hybrid computational approach that integrates evolutionary and compositional information by merging Average Block-based Position-Specific Scoring Matrix (AB-PSSM) characteristics with Dipeptide Composition (DPC) into a 600-dimensional representation. The hybrid feature space was assessed utilizing three machine learning algorithms (support vector machine, random forest, and XGBoost) alongside three deep learning architectures (CapsBiLSTM, ResCapsNetPlus, and ResNet1D) following a rigorous five-fold out-of-fold cross-validation protocol to guarantee fairness and reproducibility. A thorough assessment revealed that the hybrid attributes significantly surpassed individual descriptors, with the SVM and CapsBiLSTM classifiers attaining classification accuracies of 90% and ROC-AUC and PR-AUC values surpassing 95%. By integrating complementary evolutionary and compositional descriptors within a rigorously controlled out-of-fold validation framework, the proposed method improves predictive stability compared with previously reported sequence-based and ensemble learning predictors. Furthermore, unlike many existing approaches that primarily rely on accuracy-based evaluation, this study incorporates statistical significance testing and interpretable feature attribution, enabling both reliable generalization performance and biological interpretability. The robustness and reliability were further validated by statistical analyses, including DeLong’s test, McNemar’s test, and bootstrap confidence intervals, demonstrating that the observed enhancements were substantial and consistent. Furthermore, interpretability investigations employing SHAP feature attribution and t-SNE visualization underscored biologically significant sequence descriptors as pivotal factors influencing druggability, thus mitigating the “black box” constraint inherent in numerous deep learning methodologies. This study presents a statistically validated, interpretable, and high-performing framework for predicting druggable proteins, demonstrating competitive performance compared with existing machine learning and ensemble-based predictors while providing improved statistical reliability and interpretability, thereby establishing a clear basis for future applications in precision medicine and drug discovery.

## Introduction

Extensive protein families that are acknowledged as potential drug targets include drugs that are druggable proteins. These proteins have shown a high-affinity binding capacity to small drug-like molecules causing positive therapeutic outcomes [1, 2]. Approximately 60 percent of drug discovery initiatives fail due to a target being considered undruggable, and hence the need to identify these proteins at an early stage precisely [3]. Drug targets are important in drugs research and development and in disease therapy because the advancement of drug discovery initiatives, which requires proper identification of drug targets, depends on the druggability of a protein (Overington et al., 2006 [4]). Zhong et al. (2018) state that the process of developing a medicine and getting it to the market requires about of 10 to 15 years and costs over USD $2.5 billion. Thus, it is necessary to identify potential protein targets as soon as possible to enhance the creation of new medications. The major drug target of interest is proteins, as nearly 95 per cent of all known drug targets are proteins, and more than 92 per cent of all known drug-target interactions involve proteins [5, 6]. Some of the biochemical and structural techniques that were traditionally used included protein–protein interaction site discovery [7, 8], binding pocket estimation [9], and even ligand-based screening [10]. However, they are often costly, time consuming and dependent on expert knowledge [11]. As the large-scale proteome data and AI techniques advance, the computational pipelines have become a crucial part of the initial prioritization of drug targets (Tsuji et al., 2021 [12, 13]). Secondary structure and domain properties [14], 3D structural binding studies [15–17] have been studied; however, these attempts are limited by the limited structural representation of proteomes [18, 19].

In order to overcome these limitations, sequence-derived properties have received interest. Early machine learning (ML) systems used amino acid composition (AAC), dipeptide composition (DPC) and pseudo-amino acid composition (PseAAC) [20–22]. Yu et al. applied PROFEAT to generate 1080 features with the help of SVM and RF [8] and Chen et al. combined sequence and localisation features of ion channel targets [23]. Han et al. achieved a specific accuracy of 84 per cent using SVM with tenfold cross-validation [24]. Jamali et al. combined AAC, DPC and physicochemical properties with neural networks [25]. There were also developed network-based methodologies such as Yamanishi et al. chemical-genomic space model [26] and bipartite local model (BLM) [27] by Bleakley et al. Genetic algorithms were used to optimize the features of machine learning, such as Lin et al.’s Bagging-SVM, which achieved an accuracy of 93.78% [28]. In recent years, deep models have been applied towards sequence-based target inference, although their usefulness depends on features and rigour of evaluation [29]. Despite these developments, there are still great gaps. The majority of past studies focus on either manually generated compositional properties or structural properties without fully incorporating evolutionary data (e.g., PSSM) and composition (DPC). Many machine learning procedures are shown to perform highly without stringent validation procedures often by relying on random partitions that overstated effectiveness. There is limited research that uses out-of-fold (OOF) cross-validation or formal statistical testing to prove robustness (Ferreira et al., 2025 [30]). Also, despite similar sequence problems being solved using deep learning algorithms such as CNNs and LSTMs, their use in predicting druggable proteins has not been assessed exhaustively and they are opaque making them hard to interpret [31]. Furthermore, existing studies tend to compare ML and DL models with the same datasets and hybrid characteristics, which makes it difficult to compare them in terms of their respective advantages [32].

This paper provides a systematic prediction scheme of druggable proteins based on the hybrid model, using AB-PSSM and DPC as 600-dimensional hybrid feature representation. Accuracy, ROC-AUC, PR-AUC, F1 score, and MCC are used to evaluate the performance of models. Bootstrap confidence intervals, DeLong tests and McNemar tests also confirm the robustness [33]. Besides performance, interpretability is also emphasized by t-SNE visualization of separability of features and SHAP analysis of contribution of features [34].

*Main contributions*:

- *Hybrid feature representation*: This study proposes a novel hybrid sequence representation that combines Average Block-based Position-Specific Scoring Matrix (AB-PSSM) and Dipeptide Composition (DPC) to simultaneously capture evolutionary conservation and local sequence-order information for druggable protein prediction.
- *Fair benchmarking of machine learning and deep learning models*: The hybrid representation’s discriminative capacity is systematically evaluated using classical machine learning algorithms (SVM, Random Forest, and XGBoost) and advanced deep learning architectures (CapsBiLSTM, ResCapsNetPlus, and ResNet1D) within a unified experimental framework.
- *Rigorous evaluation and statistical validation*: This study employs a five-fold out-of-fold validation strategy complemented by statistical significance testing methods, including DeLong’s test, McNemar’s test, and bootstrap confidence interval analysis, unlike many existing studies that rely primarily on accuracy metrics, to ensure robustness and reproducibility.
- *Model interpretability and biological insight*: SHAP feature attribution and t-SNE visualization are employed for interpretability analysis to identify biologically significant sequence descriptors contributing to druggability prediction, thereby improving the transparency of deep learning models.

## Methods

### Benchmark druggable proteins dataset

In this study, we exploited benchmark dataset, initially introduced by Jamali et al. [25] and includes protein sequences that are experimentally confirmed to be both druggable and non-druggable. The positive class (druggable proteins) was picked out of the DrugBank database that originally contained 1611 proteins that had been identified to interact with drugs. The CD-HIT program has been applied with 0.5 sequence identity cutoff to reduce redundancy of the sequence and also ensure high-quality training data. The positive class After filtering and alignment between the sets of features, we kept 1218 unique druggable sequences of protein in the positive class For the negative class, we followed the methodology of Bakheet et al. [36] and Li et al. [21]. Protein sequences were retrieved from the Swiss-Prot database and were processed on the CD-HIT to eliminate redundancy. The above resulted in the development of 1319 non-druggable protein sequences that comprised the negative class.

After preprocessing and integrating, the resulting final benchmark dataset comprised 2537 proteins, comprising 1218 and 1319 druggable and non-druggable proteins, respectively. This filtered data were consistently used in all subsequent feature mining and model testing studies, which ensured consistency and equality (Table 1).

**Table 1:** Druggable and non-druggable proteins in benchmark and independent datasets

Dataset	Druggable proteins	Non-druggable proteins	Total
Benchmark (Before CD-HIT)	1611	1224	2835
Final (After CD-HIT, 0.5 cut-off)	1218	1319	2537

### Feature extraction using dipeptide composition (DPC)

Dipeptide Composition (DPC) is a basic feature extraction technique that we used to combine sequence-order data in characterizing proteins. DPC is biologically meaningful encoding of sequence patterns which facilitates interpretation of the structure and functions of proteins in that it encodes the local correlation between the two sequential amino acids.

Given a protein sequence of length the value of each dipeptide can be calculated as:

1 $$ DPC(i) = \frac{{N_{i} }}{L - 1} $$

where $N_{i}$ indicates how many times the iii-th dipeptide appears in the protein sequence.

The simplicity and efficiency of DPC in reflecting local sequence features has led to its wide application in several bioinformatics applications, including structural class prediction, protein subcellular localisation and drug-target identification [37–39]. The analysis of DPC features of FASTA- format protein sequences was performed by using the i Feature Omega command-line software. These features have then been added to AB-PSSM features to form a hybrid input representation that would be used in deep learning-based classification.

### Feature extraction using average block-based PSSM (AB-PSSM)

To incorporate evolutionary information into the feature representation we used the Average Block-based Position-Specific Scoring Matrix (AB-PSSM) method. To extract homologous sequence pattern conservation of amino acids, bioinformatics experts often use the Position-Specific Scoring Matrix (PSSM) which forms the basis of the fixed-length feature transformation procedure called AB-PSSM [40, 41]. PSI-BLAST was repeated three times with the Swiss-Prot database, and PSSM profiles of the protein sequence were generated with an E-value cutoff of 0.001. All PSSMs are L×20 size matrices and L is the length of the protein chain and 20 is the standard amino acids.

To convert this variable-length matrix into machine and deep learning models, we applied the AB-PSSM method to convert it into a fixed-length matrix. All the PSSMs were divided into 10 equal length sequence segments. Each block had an average score for each amino acid column.

2 $$ AB\_PSSM(b,a) = \frac{1}{{|B_{b} |}}\sum\limits_{{i \in B_{b} }} {PSSM(i,a)} $$

where:

PSSM $(i,a)$ is the PSSM score at position $i$ for amino acid $b$,

$B_{b}$ denotes the set of sequence positions in block $b$,

AB_PSSM $(b,a)$ is the average score of amino acid a in block $b$.

Besides sequence-composition descriptors such as DPC, this 200-dimensional AB-PSSM vector also works well in capturing localised evolutionary trends. The method combines evolutionary conservation and local sequence-order input in our hybrid representation and improves the discriminative ability to predict druggable proteins [42, 43].

### Hybrid features (AB-PSSM + DPC)

To combine the advantages of the discriminative power of the descriptors, we designed a hybrid feature representation with a combination of Dipeptide Composition (DPC) and Average Block-based PSSM (AB-PSSM). AB-PSSM stores patterns of evolutionary conservation acquired using homologous sequences, whilst DPC depicts local sequence-order correlations between adjacent residues. Even though individually each of the descriptors provides good biological information, their combination is expected to capture the complementary nature of proteins.

Formally, if $F_{AB - PSSM} \in {\mathbb{R}}^{200}$ signifies the 200-dimensional vector derived from AB-PSSM and $F_{DPC} \in {\mathbb{R}}^{400}$ symbolises the 400-dimensional vector from DPC, the hybrid representation is articulated as:

3 $$ F_{Hybrid} = F_{AB - ossm} ||F_{DPC} $$

In which “||” denotes the concatenation of vectors, representing the integration of compositional and evolutionary context. Similar feature fusion algorithms have shown stronger prediction capability in protein bioinformatics use cases [44].

### Proposed method (step-by-step workflow)

**Fig. 1:**
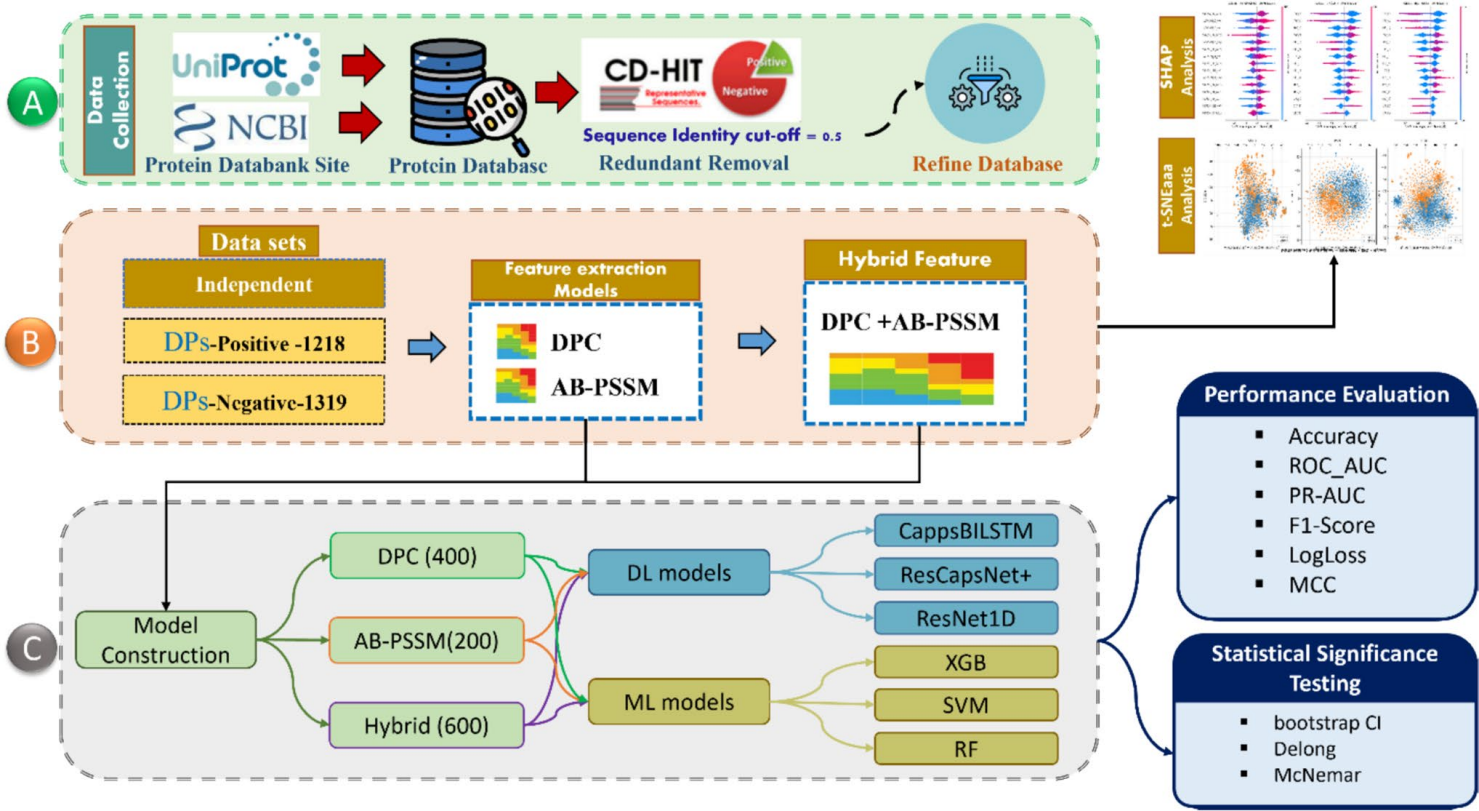
Overall workflow of the proposed hybrid framework for druggable protein prediction

The classification system described in this paper is a deep hybrid system following a structured pipeline as shown in Fig. 1. The process is divided into four major steps that include dataset preparation, feature extraction, model training, and statistical validation.

The initial one was the development of a benchmark dataset comprising of druggable and non-druggable proteins. The DrugBank database was used to obtain druggable proteins and Swiss-Prot to obtain non-druggable proteins to facilitate diversification to reduce redundancy. This was done using CD-HIT with sequence identity threshold of 0.5. The number of obtained preprocessed dataset sequences amounted to 2537 and contained 1218 and 1319 druggable and non-druggable proteins respectively. This data has been corroborated again using disease-related protein collections, 230 essential proteins of a breast cancer study [45], 2353 cancer driver genes of the Network of Cancer Genes [46], and 1365 RNA-binding proteins.

In the second step, reading of proteins was changed into a number format. There were obtained two complementary descriptions. Dipeptide Composition (DPC) is a measure of the frequency of 400 pairs of amino acids that represents local sequential dependencies. AB-PSSM divides the data into 10 blocks to outline the patterns of conserved evolutionary features by averaging the log-odds substitution scores of amino acids to create 200-dimensional vectors. We have developed a 600-dimensional hybrid feature (a concatenation of these descriptors) that consists of both sequence-order and evolutionary information.

The predictive modelling was then done using hybrid attributes. Three classical machine learning algorithms were evaluated Support Vector Machine (SVM) [47], Random Forest (RF) [48], and Extreme Gradient Boosting (XGBoost) [49].

The performance was well evaluated based on a number of complementary measures, such as accuracy, ROC-AUC, PR-AUC, F1-score, MCC, precision, recall, and log-loss. Along with the prediction accuracy, statistical rigour was also ensured by bootstrap confidence interval, DeLongs test and McNemar test. Finally, the interpretability was highlighted with SHAP analysis that quantified feature contributions, and t-SNE visualisation that was used to depict the feature separability in a low-dimensional setting. The elaborated flowchart of the proposed methodology (Fig. 1) shows the combination of dataset curation, hybrid feature extraction, ML/DL benchmarking, statistical validation, and interpretability into a whole process of predicting drugs to act on a particular protein.

### Predictive models

To fully test the discriminative ability of the hybrid feature representation, we used three machine learning algorithms and three deep learning architectures. Machine learning area included the Support Vector machine (SVM) [47], Random Forest (RF) [48], and the Extreme Gradient Boosting (XGBoost) [49] that are widely used due to their strength, scalability, and high performance when used in categorising sequences of high-dimensionality. The Residual Capsule Network Plus (ResCapsNetPlus) residual learning Directional extension of capsule routing with better hierarchical representation and ResNet1D, a type of residual networks applied to one-dimensional biological sequences modelling [50–52].

### Model training and evaluation protocol

Five different folds in the dataset were used and each fold was used in turns as a test set and the rest had been used as training dataset. The performance was assessed using different measures: Accuracy, ROC-AUC, PR-AUC, F1-score, Matthews Correlation Coefficient (MCC), Precision, Recall, and Log-loss and made it possible to evaluate the predictive reliability in a comprehensive way. T-tests Bootstrap confidence intervals, DeLong test of ROC curves and McNemar test of classifier pairs were used to further verify statistical strength.

Early stopping and dropout regularisation were used to alleviate overfitting in deep models training. The Adam optimiser that used modified learning rates was employed to optimise. Every implementation was done using Python 3.10, and scikit-learn machine learning models and PyTorch deep learning models were used.

### Experimental setup and hyperparameter configuration

All machine learning and deep learning models were implemented under a unified experimental framework. A stratified five-fold out-of-fold (OOF) cross-validation strategy was employed, and identical data splits were reused across all models to ensure fair comparison. All hyperparameters were finalized based on validation performance within training folds to avoid information leakage and ensure fair model comparison. Feature scaling was performed within each training fold only to prevent data leakage. The detailed hyperparameter configurations of deep learning models are summarized in Table 2. To reduce potential overfitting caused by the moderate dataset size, dropout regularization, early stopping, and stratified five-fold cross-validation were used during model training, while machine learning model hyperparameters were optimized within the same cross-validation framework.

**Table 2:** Hyperparameter configuration of deep learning models

Hyperparameter	CapsBiLSTM	ResCapsNetPlus	ResNet1D
Optimizer	Adam	Adam	Adam
Learning rate	0.001	0.001	0.0003
Batch size	64	64	128
Epochs	60	60	60
Dropout rate	0.2	0.2	0.2
Activation (hidden / output)	ReLU / Softmax	ReLU / Softmax	ReLU / Softmax

The scikit-learn framework was used to create the machine learning models, which included Support Vector Machine (SVM), Random Forest (RF), and Extreme Gradient Boosting (XGBoost), all using the same cross-validation protocol. The SVM model evaluated both linear and radial basis function (RBF) kernels, optimizing the regularization parameter (C) and kernel coefficient (γ) by grid search inside training folds. The Random Forest classifier was created utilizing 1000 decision trees with a maximum depth of 8 to optimize model complexity and generalization capabilities. The XGBoost model employed gradient boosting decision trees with a maximum depth of 2, a learning rate of 0.03, and a subsampling ratio of 0.6. To ensure repeatability and equitable comparison across models, identical stratified five-fold cross-validation splits with a fixed random seed were used to train all machine learning models.

## Model performance evaluation

All of the models were evaluated based on the measures of the confusion matrix, including Accuracy, Precision, Recall, and F1-score. These metrics are obtained based on the confusion matrix which includes true positive (TP), true negative (TN), false positive (FP) and false negative (FN). Accuracy is determined as the proportion of the incidences of classification that are correct to the number of samples.

4 $$ Accuray = \frac{{T_{P} + T_{n} }}{{T_{p} + T_{n} + F_{p} + F_{n} }} $$

5 $$ \Pr ecision = \frac{{T_{P} }}{{T_{p} + F_{p} }} $$

6 $$ {\mathrm{Re}} call = \frac{{T_{P} }}{{T_{p} + F_{n} }} $$

7 $$ F_{1} - Score = 2 \times \frac{\Pr ecision \times recall}{{\Pr ecision + recall}} $$

Precision-Recall (PR) curves and area under the PR curve (AUPRC) in particular are informative as well as compared to ROC-AUC, which might have a disproportionately positive evaluation in class-imbalanced data. Therefore, to provide a complete evaluation of model effectiveness, both ROC-AUC and AUPRC were included.

## Results and discussion

The aim of this work was to introduce new and reliable classification algorithms to predict the druggable proteins. We have done this by considering two free protein sequence descriptors: the dipeptide composition (DPC) and average block-based position-specific scoring matrix (AB-PSSM) and its hybrid representation. The different complementing metrics used to evaluate the model performance such as Accuracy, F1-score, MCC, ROC-AUC, PR-AUC were used to enable a comprehensive comparison between the respective benefits of the ML and DL methodologies.

### Performance across models

**Fig. 2:**
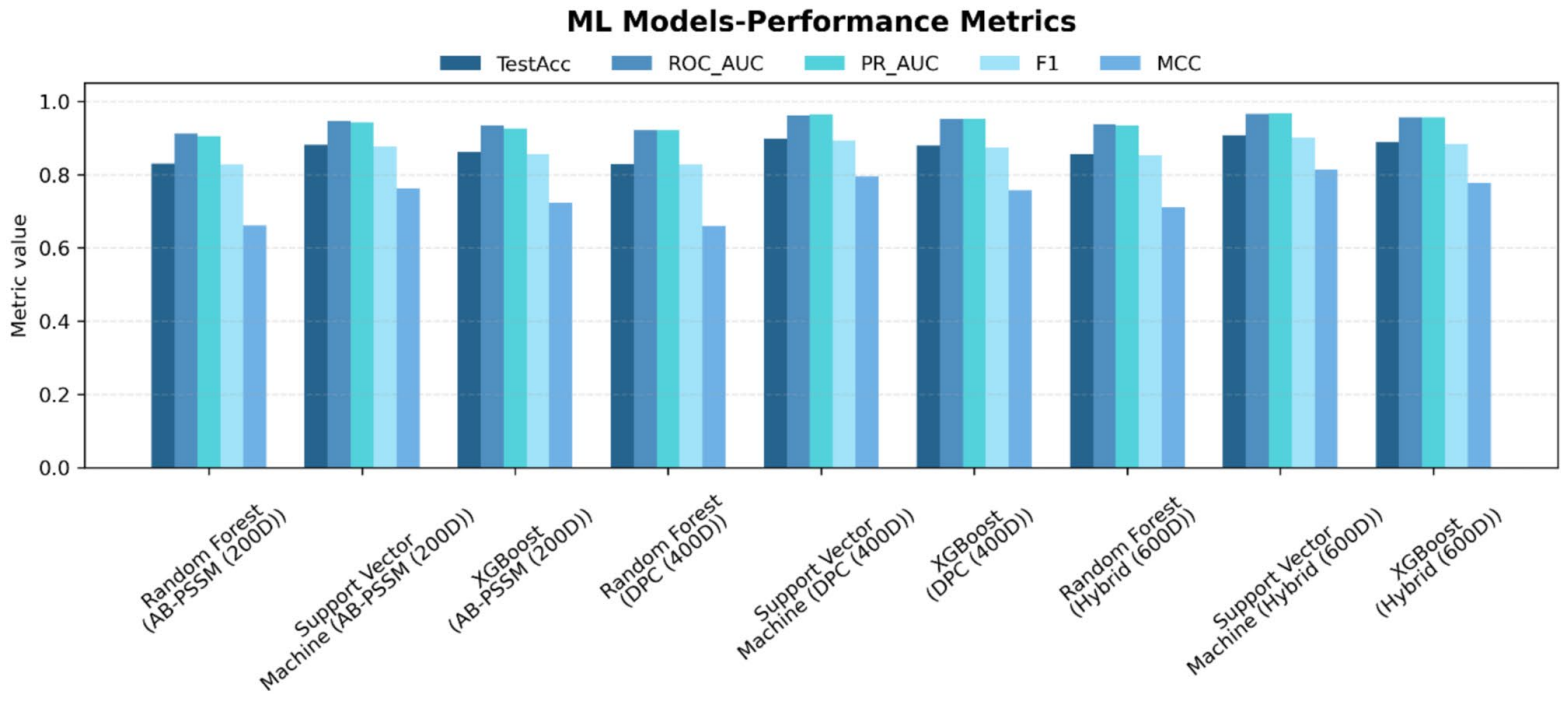
ML models visual comparison of classifier performance using evaluation metrics

Table 3 and Figs. 2 (ML models) and 3 (DL models) indicate the mean five-fold out of fold (OOF) results across all classifiers and feature representations. Both deep learning and machine learning models demonstrated that they were able to make proper predictions. In applying the hybrid 600D functionality as opposed to the single descriptors (AB- PSSM 200D or DPC 400D), the models were always doing better. The most overall best ML model was the Support Vector Machine (SVM) with the ROC-AUC of 0.966 and PR-AUC of 0.967 using the hybrid features. SVM also demonstrated tremendous generalisation and inter-fold stability, demonstrating the degree of strong-kernel approaches to high-dimensional biological data. XGBoost was not behind far behind, particularly with the hybrid and DPC feature sets. Random Forest received much lower scores, though it was competitive.

**Table 3:** Overall five-fold OOF performance across ML and DL models using AB-PSSM, DPC, and Hybrid (600D) features

Model	Feature	TrainAcc	Accuracy	ROC-AUC	PR-AUC	F1	Precision	Recall	MCC	LogLoss
SVM	AB-PSSM	95.211	88.148	94.612	94.206	87.692	87.622	87.794	0.763	0.289
SVM	DPC	93.999	89.752	96.078	96.447	89.343	89.208	89.492	0.795	0.254
SVM	Hybrid	97.822	90.658	96.556	96.719	90.202	90.865	89.575	0.813	0.237
RF	AB-PSSM	90.75	82.998	91.255	90.525	82.758	80.665	85.004	0.661	0.464
RF	DPC	90.658	82.933	92.085	92.107	82.737	80.347	85.307	0.66	0.448
RF	Hybrid	92.521	85.534	93.76	93.495	85.253	83.491	87.112	0.711	0.437
XGB	AB-PSSM	93.756	86.178	93.422	92.664	85.674	85.335	86.045	0.723	0.325
XGB	DPC	95.851	87.899	95.174	95.293	87.37	87.508	87.277	0.758	0.289
XGB	Hybrid	96.029	88.845	95.721	95.698	88.419	88.101	88.754	0.777	0.273
ResNet1D	AB-PSSM	82.63	79.806	88.679	86.24	80.179	76.168	85.686	0.61	0.477
ResNet1D	DPC	79.286	77.493	86.476	84.345	76.469	77.411	76.77	0.558	0.477
ResNet1D	Hybrid	84.282	81.318	90.132	87.804	82.464	75.782	90.883	0.645	0.457
ResCapsNetPlus	AB-PSSM	94.445	87.177	93.352	92.627	86.844	85.776	88.038	0.744	0.506
ResCapsNetPlus	DPC	95.546	87.703	94.146	94.469	87.174	87.324	87.11	0.754	0.504
ResCapsNetPlus	Hybrid	96.393	89.161	95.366	95.382	88.833	88.008	89.735	0.784	0.493
CapsBiLSTM	AB-PSSM	91.975	88.122	94.158	93.327	87.750	87.261	88.313	0.763	0.482
CapsBiLSTM	DPC	92.107	90.027	95.592	95.996	89.482	90.678	88.340	0.800	0.469
CapsBiLSTM	Hybrid	93.683	90.500	96.284	96.526	90.172	89.550	90.804	0.810	0.469

**Fig. 3:**
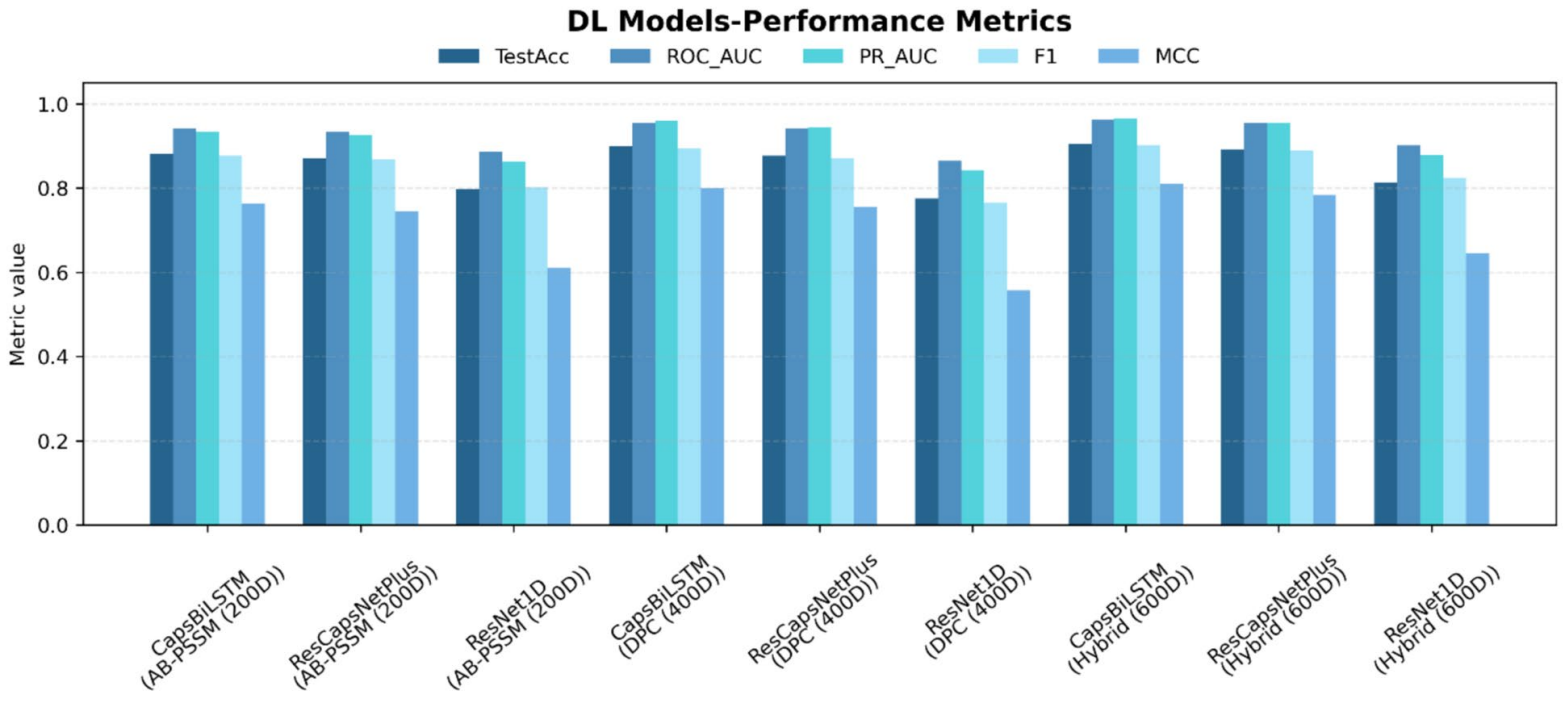
DL models visual comparison of classifier performance using evaluation metrics

CapsBiLSTM and ResCapsNetPlus invariably outperform the ResNet1D in the DL family. The PR-AUC and ROC-AUC of CapsBiLSTM (Hybrid 600D) were 0.964 and 0.963 respectively which is very similar to SVM. ResCapsNetPlus performed well also on all measures. On the other hand, some of the deep learning models exhibited an overfitting behavior as the training accuracy was better than the test accuracy of the model. This implies that they were not as stable as ML models.

Machine learning (SVM) based on kernels functioned somewhat better and were more stable, whereas capsule-based deep learning (DL) matched the accuracy and provided originality to the dependence on protein sequences.

### Confusion matrix analysis

**Fig. 4:**
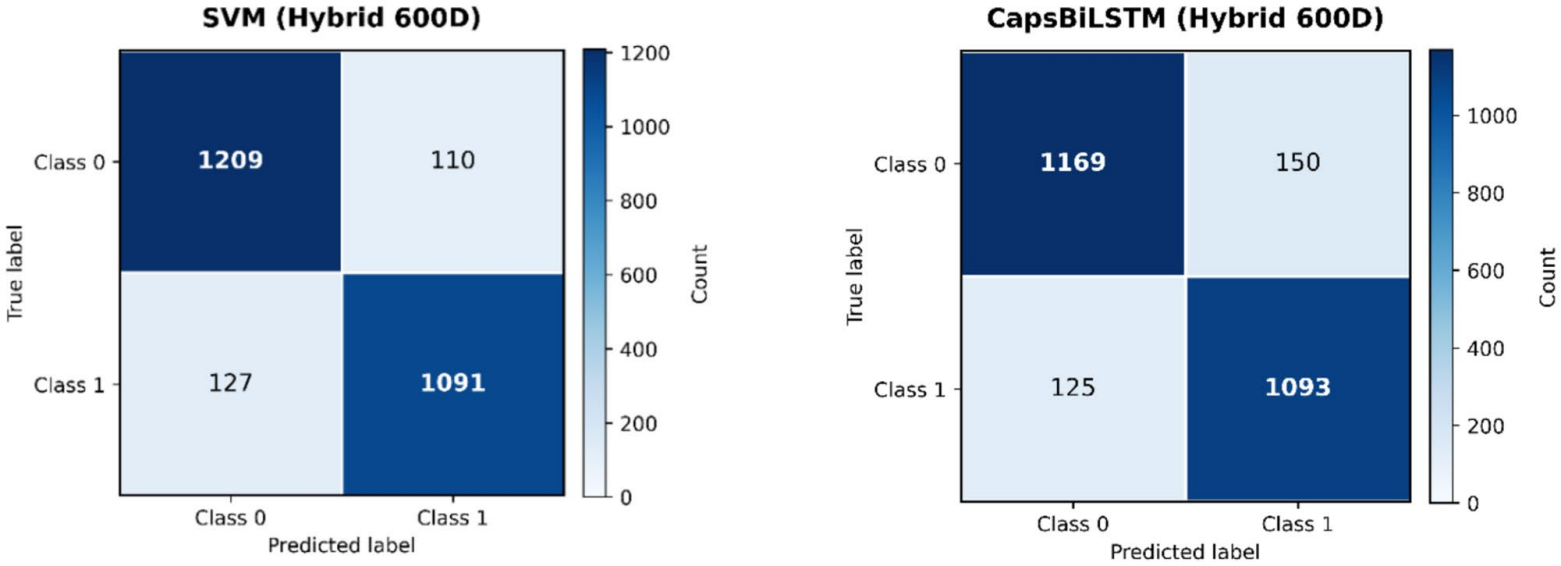
Confusion matrices of top classifiers (SVM and CapsBiLSTM) using Hybrid (600D) features

The visualisation of confusion matrices of two classifiers with the highest performance, SVM (Hybrid 600D) and CapsBiLSTM (Hybrid 600D), was used to have a more detailed evaluation of the models behaviour (Fig. 4). Such matrices, in turn, demonstrate the distribution of true positives (TP), true negatives (TN), false positives (FP), and false negatives (FN) and, consequently, represent a detailed analysis of the effectiveness of each model in distinguishing between druggable and non-druggable proteins.

CapsBiLSTM (Hybrid 600D) model correctly predicted 1169 non-druggable proteins with 1093 druggable proteins, with the false positives were 150 and false negatives were 125. This demonstrates strong categorisation ability, however, the slight rise in false positive indicates a tendency to be more liberal in its anticipations of positive cases. The SVM (Hybrid 600D) on the other hand was the highest performing with 1209 true negatives and 1091 true positives with only 110 instances of false negativity and 127 instances of false positivity. This outcome implies a reduction in errors, an increase in overall stability, and an increase in the equity of classifications between positive and negative groups. These confusion matrix visualisations support the finding that SVM is the most fair in terms of classification results, despite CapsBiLSTM remaining highly competitive and generally applicable. The models depict that the hybrid 600D representation is far much better than single-feature descriptors in terms of discriminative performance.

### ROC curve evaluation

**Fig. 5:**
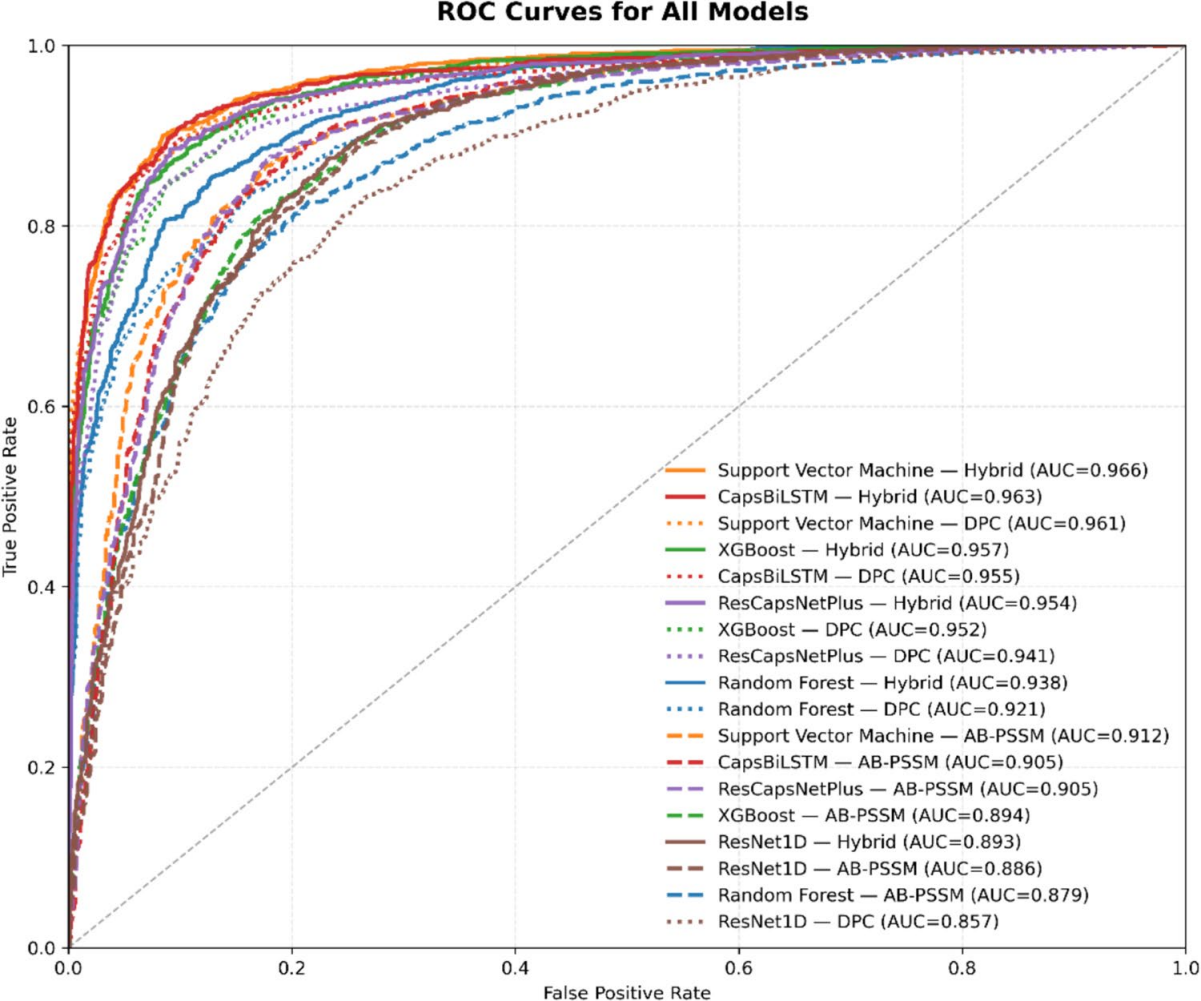
ROC curves (5-fold OOF) for all models across feature sets

The efficacy of each of the classifiers was assessed using Receiver Operating Characteristic (ROC) curves to compare the performance of the classifiers in differentiating between druggable and non-druggable proteins. These graphs demonstrate the true positive rate (TPR) relative to the false positive rate (FPR) across varying decision levels that provide a comprehensive view of model sensitivity and specificity. Figures 5 (all models), 6 (ML models only), and 7 (DL models only) illustrate the ROC curves that were obtained by five-fold out-of-fold (OOF) validation. As shown in Fig. 5, both ML and DL models achieved high ROC-AUC scores, and therefore confirming the strong predictive ability of the proposed framework. Support Vector machine (Hybrid 600D) was the best machine learning algorithm with an Area Under the Curve (AUC) of 0.966 and XGBoost (Hybrid 600D) with an AUC of 0.957. Random Forest achieved slightly worse performance (AUC = 0.938) but is able to establish competitive results at any set of features. CapsBiLSTM (Hybrid 600D) achieved a ROC-AUC of 0.963 in the DL group, and this is closely comparable to SVM, but ResCapsNetPlus (Hybrid 600D) also showed high performance with a ROC-AUC of 0.954. Comparatively, ResNet1D had lower values of AUC, which represents a reduced discriminatory power.

**Fig. 6:**
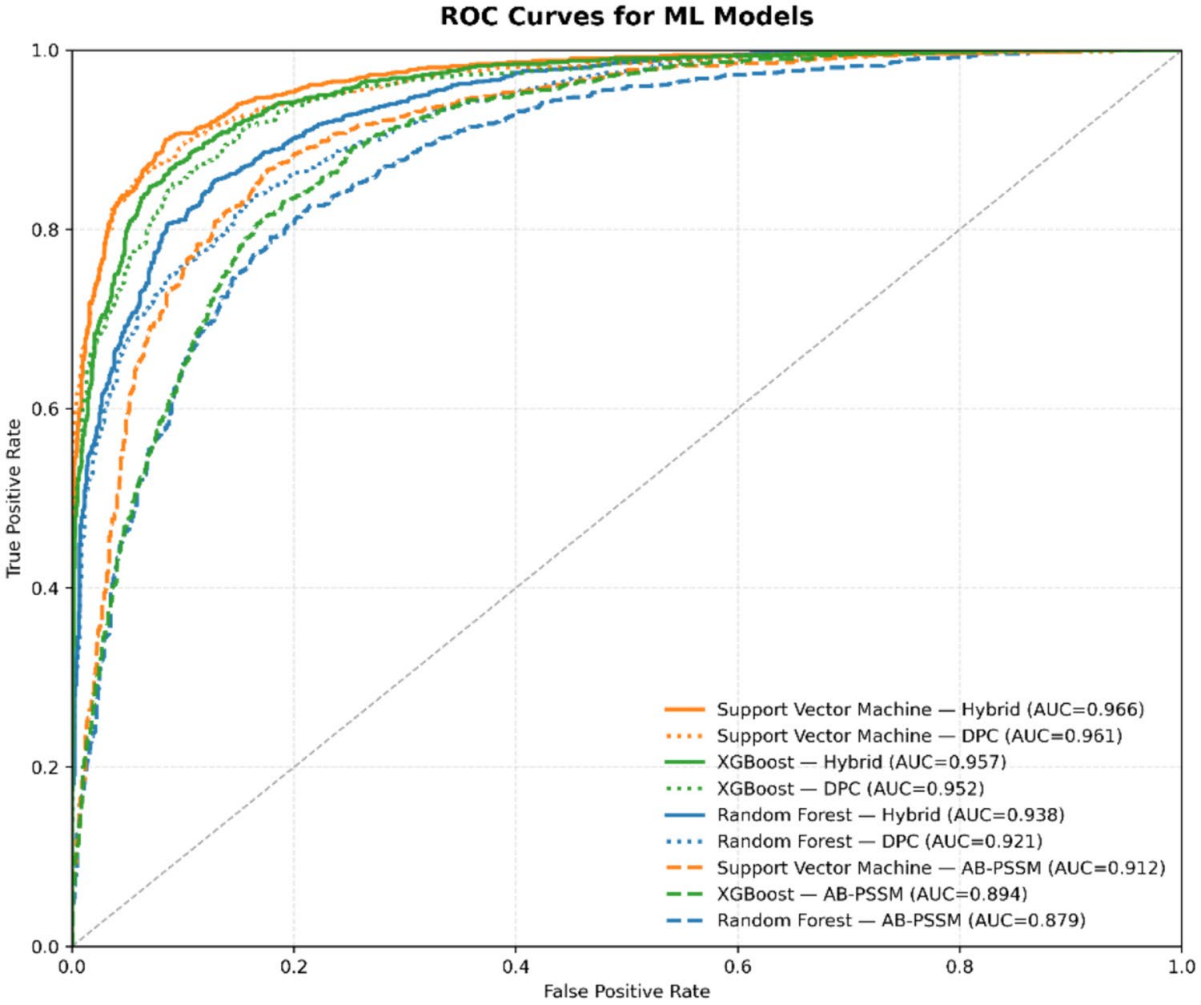
ROC curves (5-fold OOF) for machine learning models

**Fig. 7:**
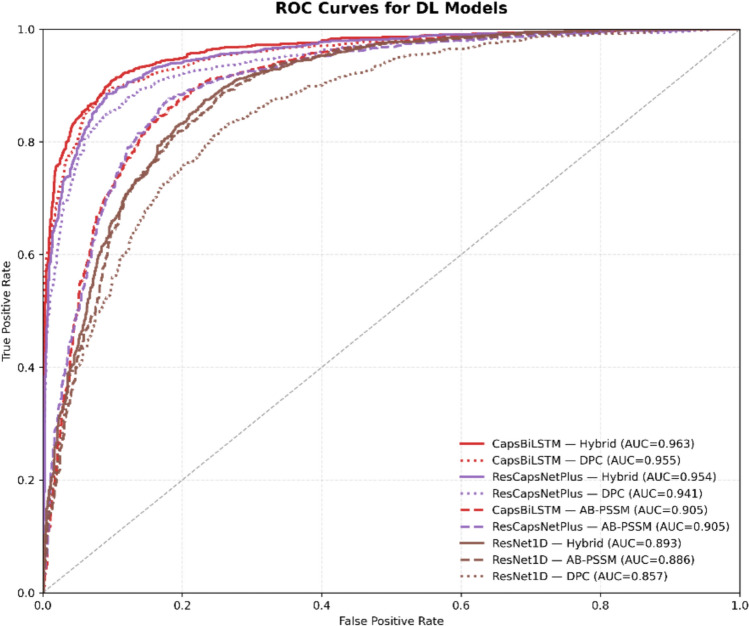
ROC curves (5-fold OOF) for deep learning models only

There are comparisons within families as shown in Figs. 6 and 7. Capsule-based structures (CapsBiLSTM and ResCapsNetPlus) outperformed ResNet1D in deep learning, which demonstrates the power of capsule mechanisms to address sequence dependencies. Support Vector Machines (SVM) demonstrated the most stable and consistent behavior with all feature sets, in particular, the hybrid one, in the context of machine learning. Conversely, XGBoost became a viable and efficient competitor, whereas the performance of the Random Forest was slightly inferior.As it has been shown in the ROC curve analysis, fusion of hybrid features enhances the classification performance significantly, and the best models are SVM and CapsBiLSTM, which have the most appropriate balance between sensitivity and specificity. These findings are in line with the conclusions reached through overall performance measures and confusion matrices.

**Fig. 8:**
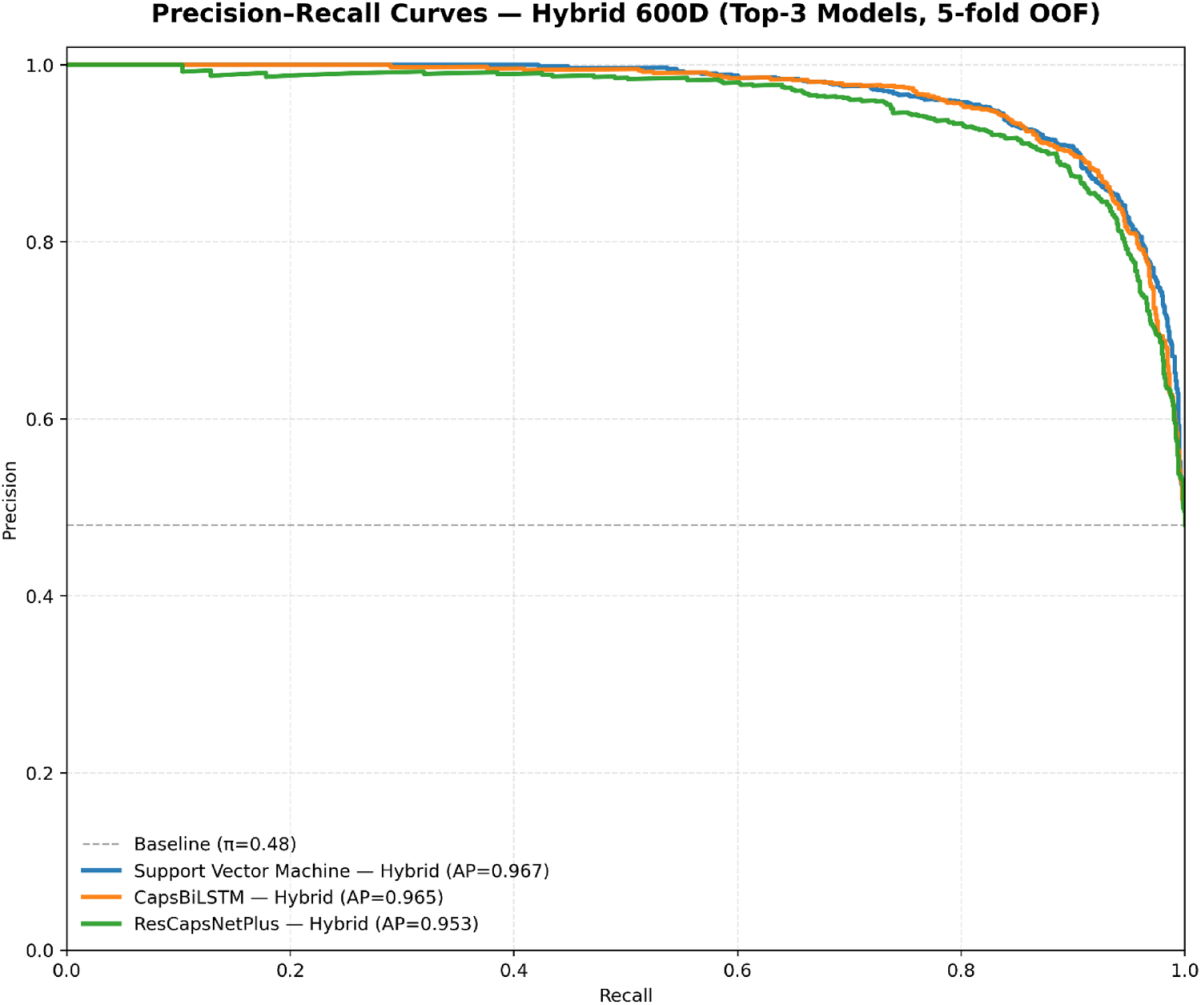
Precision-Recall curves for the top three classifiers trained on Hybrid 600D features under five-fold out-of-fold validation

ROC curves measure the overall discriminative ability, but Precision-Recall (PR) curves give a complementary picture by focusing on the quality of positive-class predictions. Although the dataset is relatively balanced, being a biomedical use, PR curves are highly essential because false positives may have misleading implications on further analysis. We have analysed the PR curves of the top 3 classifiers, namely SVM, CapsBiLSTM, and ResCapsNetPlus, trained on Hybrid 600D features, using five-fold out-of-fold (OOF) validation (Fig. 8, Table 4). The results of the analysis have shown that all three models had steady and high performance levels, but SVM delivered the highest area under PR curve (AP = 0.967) and CapsBiLSTM (AP = 0.965) and ResCapsNetPlus (AP = 0.953).

**Table 4:** Precision-Recall curve results (AP values) for the top three classifiers trained on Hybrid 600D features under five-fold OOF validation

Model	Feature	AP (AUC-PR)
Support vector machine (SVM)	Hybrid 600D	0.967
CapsBiLSTM	Hybrid 600D	0.965
ResCapsNetPlus	Hybrid 600D	0.953

The results support the conclusions derived from the ROC analysis. The combination of AB-PSSM and DPC features in a hybrid representation significantly enhances classification reliability, with SVM and CapsBiLSTM identified as the most effective frameworks for druggable protein identification. The consistently elevated AP values presented in Table 4 indicate that these models effectively identify true druggable proteins while reducing false positives, thereby affirming their reliability and applicability in predictive drug discovery. Random Forest followed significantly behind XGBoost, which was evaluated as a strong alternative across all feature sets, especially with the hybrid representation.

The ROC curve analysis indicates that hybrid feature fusion markedly improves classification performance, with SVM and CapsBiLSTM identified as the most effective models, demonstrating an optimal balance between sensitivity and specificity. The results support the conclusions derived from comprehensive performance metrics and confusion matrix analyses.

### Statistical significance and robustness

The performance differences between competing classifiers are frequently not statistically significant, despite the fact that standard metrics like accuracy, precision-recall, and ROC curves are frequently provided. We integrated formal statistical testing and uncertainty estimation into the evaluation pipeline to enhance the robustness of our findings. We employed DeLong’s nonparametric test for correlated ROC curves to assess discriminative performance, McNemar’s test for correlated proportions to examine misclassification patterns, and bootstrap resampling to quantify variability in secondary metrics including PR-AUC, F1 score, and MCC. Furthermore, we evaluated model calibration using Brier scores and reliability curves [53], and we analysed operating point stability at strict thresholds (precision or recall ≥ 0.90). This study integrates complementary analyses to extend beyond mere accuracy values, offering a robust statistical framework for interpreting model performance in druggable protein prediction.

#### Pairwise AUC comparisons (DeLong test)

The initial phase of our statistical analysis involved assessing the significance of differences in ROC-AUC between models through DeLong’s test [54], a nonparametric method for comparing correlated ROC curves from the same dataset. Pairwise comparisons were conducted among the three primary classifiers: SVM, CapsBiLSTM, and ResCapsNetPlus. The results presented in Table 5 demonstrate that SVM and CapsBiLSTM attained comparable ROC-AUC values of 0.967 and 0.965, respectively, resulting in a ΔAUC of − 0.002 and a 95% confidence interval spanning from − 0.006 to 0.002. The observed difference was not statistically significant, indicating that both models exhibit similar discriminative capacity. However, ResCapsNetPlus consistently underperformed, achieving a ROC-AUC of 0.953 and demonstrating significant deficits of − 0.014 compared to SVM and − 0.012 compared to CapsBiLSTM. The findings indicate that SVM and CapsBiLSTM constitute a statistically indistinguishable top-performing group, whereas ResCapsNetPlus, despite being competitive, is positioned in a lower tier of predictive accuracy. This analysis highlights that the superiority of SVM compared to CapsBiLSTM lacks statistical support, emphasising the necessity of cautious interpretation of minor differences in headline metrics.

**Table 5:** Pairwise AUC comparisons using DeLong’s test among SVM, CapsBiLSTM, and ResCapsNetPlus (Hybrid 600D)

Metric	SVM vs ResCapsNetPlus	CapsBiLSTM vs ResCapsNetPlus	SVM vs CapsBiLSTM
AUC_SVM	0.965735	0.965735	0.965735
AUC_CapsBiLSTM	0.96285	0.96285	0.96285
AUC_ResCapsNetPlus	0.953897	0.953897	0.953897
∆AUC	0.011838	0.008953	0.002885
95% CI	[0.007011, 0.016665]	[0.004094, 0.013812]	[− 0.001282, 0.007053]
p (raw)	1.53E-06	0.000305	0.1748
p (Holm)	4.60E-06	0.00061	0.1748
Significant (α = 0.05)	Yes	Yes	No

#### Error distribution comparisons (McNemar’s test)

We utilized McNemar’s test [55], which determines if two classifiers disagree systematically on particular prediction outcomes, to further analyze any variations in misclassification patterns. The test evaluates discordant counts, represented as cases misclassified by the first model but accurately classified by the second, and vice versa, so determining if one classifier consistently rectifies the errors of another. The comparison of SVM and CapsBiLSTM demonstrated approximately equivalent discrepancies, with no statistically significant difference in their error distributions (Table 6). This signifies that both models attain comparable discriminative accuracy, as demonstrated by DeLong’s test, while also incurring significantly overlapping classification mistakes. Conversely, pairwise comparisons of ResCapsNetPlus revealed asymmetries, indicating that its predictions more frequently deviate from the superior models. These findings substantiate the assertion that SVM and CapsBiLSTM constitute a statistically indistinguishable cohort of top performers, while ResCapsNetPlus exhibits unique and less dependable error patterns aligned with its inferior AUC performance.

**Table 6:** Error distribution comparisons using McNemar’s test between top-performing classifiers (Hybrid 600D)

Metric	SVM vs CapsBiLSTM	SVM vs ResCapsNetPlus	CapsBiLSTM vs ResCapsNetPlus
Threshold	0.5	0.5	0.5
n01 (A wrong, B right)	70	70	66
n10 (A right, B wrong)	74	108	100
X^2^ (corr)	0.0625	7.691	6.5602
P	0.8026	0.00555	0.01043
Favored	SVM	SVM	CapsBiLSTM

#### Metric stability via bootstrap confidence intervals

To quantify the uncertainty linked to secondary evaluation metrics and assess the robustness of observed differences against sampling variability, we utilized a bootstrap resampling strategy [56]. Out-of-fold predictions underwent resampling 10,000 times with replacement, and for each replicate, the PR-AUC, F1 score, and Matthews correlation coefficient (MCC) were recalculated. Median values and bias-corrected 95% confidence intervals were derived from these distributions for each model (Table 7).

**Table 7:** Bootstrap estimates of PR-AUC, F1-score, and MCC with 95% confidence intervals for SVM, CapsBiLSTM, and ResCapsNetPlus (Hybrid 600D)

Metric	SVM	CapsBiLSTM	ResCapsNetPlus
PR-AUC (median [95% CI])	0.967 [0.961, 0.973]	0.965 [0.958, 0.971]	0.953 [0.944, 0.962]
F1 (median [95% CI])	0.902 [0.889, 0.914]	0.902 [0.889, 0.914]	0.888 [0.875, 0.901]
MCC (median [95% CI])	0.813 [0.790, 0.835]	0.810 [0.787, 0.833]	0.783 [0.759, 0.807]
∆PR-AUC vs SVM (median [95% CI])	–	− 0.002 [− 0.006, 0.002]	− 0.014 [− 0.020, − 0.008]
∆F1 vs SVM (median [95% CI])	–	− 0.000 [− 0.010, 0.009]	− 0.014 [− 0.024, − 0.003]
∆MCC vs SVM (median [95% CI])	–	− 0.003 [− 0.021, 0.015]	− 0.030 [− 0.050, − 0.009]

The results indicated that SVM attained the highest performance across all three metrics, exhibiting a median PR-AUC of 0.967 [95% CI: 0.961–0.973], an F1 score of 0.902 [0.889–0.914], and an MCC of 0.813 [0.790–0.835]. CapsBiLSTM demonstrated comparable performance, as evidenced by overlapping confidence intervals across all instances, indicating that the two models are statistically indistinguishable. In contrast, ResCapsNetPlus exhibited lower values, especially for MCC (0.783 [0.759–0.807]), with confidence intervals that did not overlap with those of SVM. The bootstrap analysis confirmed that the narrow uncertainty ranges (± 0.01–0.02) indicate high stability of the results, and that the relative ranking of the models is consistent across resampling. The findings demonstrate that SVM and CapsBiLSTM constitute a statistically equivalent group of top-performing models. In contrast, ResCapsNetPlus, while competitive, exhibits lower reliability when assessed using resampling-based uncertainty estimates. The bootstrap confidence intervals offer further support for the robustness and reproducibility of the classification results.

### Interpretability results

**Fig. 9:**
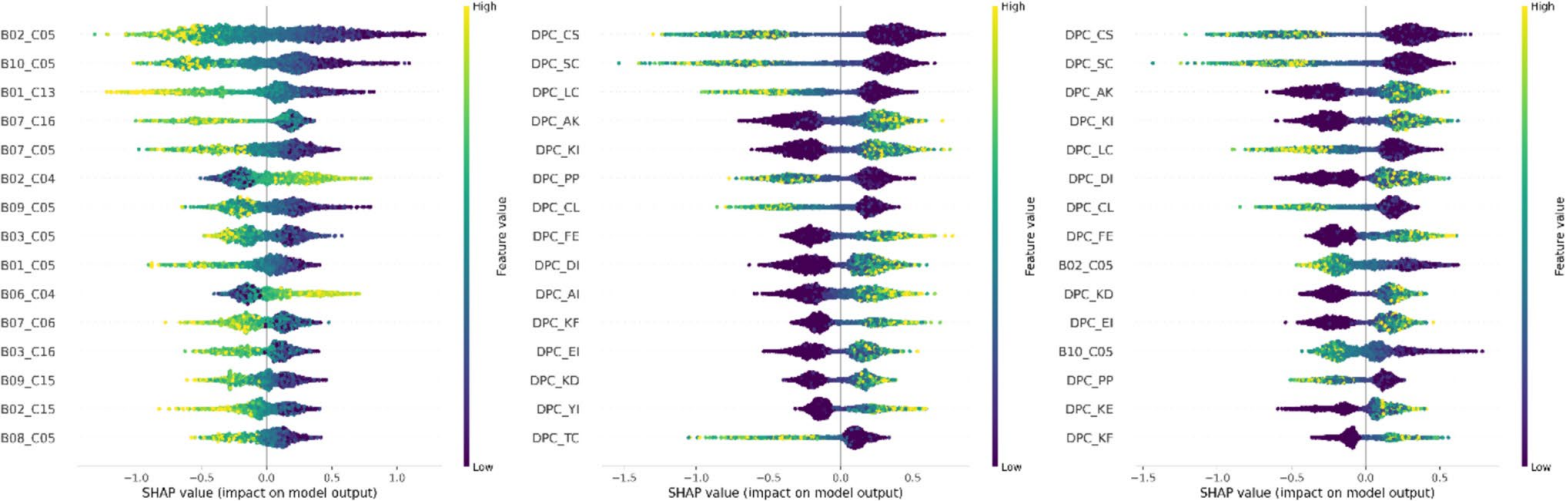
SHAP beeswarm plots of feature importance across AB-PSSM, DPC, and Hybrid (600D) representations

Interpretability analysis was conducted to clarify the factors influencing model predictions and to assess the biological validity of the derived descriptors. To display feature distributions in reduced dimensional space [61], we used t-SNE (t-distributed stochastic neighbor embedding), and to quantify the contribution of individual features [54], we used SHAP (SHapley Additive exPlanations). The complementary approaches offer feature-level attribution and global representation analysis, thus improving the transparency of the proposed framework.

(Fig. 9) Block-position descriptors such as B02C05, B10C05, and B01C13 in the AB-PSSM representation were found to have significant contributions to the classification results repeatedly which supports the relevance of local evolutionary signals. As shown in the DPC representation, there was predominance in the attribution profiles with dipeptide combinations like DPCCS, DPCSC and DPCAK indicating the importance of the sequence-order information in establishing druggability. The Hybrid 600D was revealed to present the combination of the two sets of descriptors, and DPCCS and B02C05 were found to be the most significant ones, hence ensuring that the fused representation is skillfully capturing the complementary patterns derived by sequences.

**Fig. 10:**
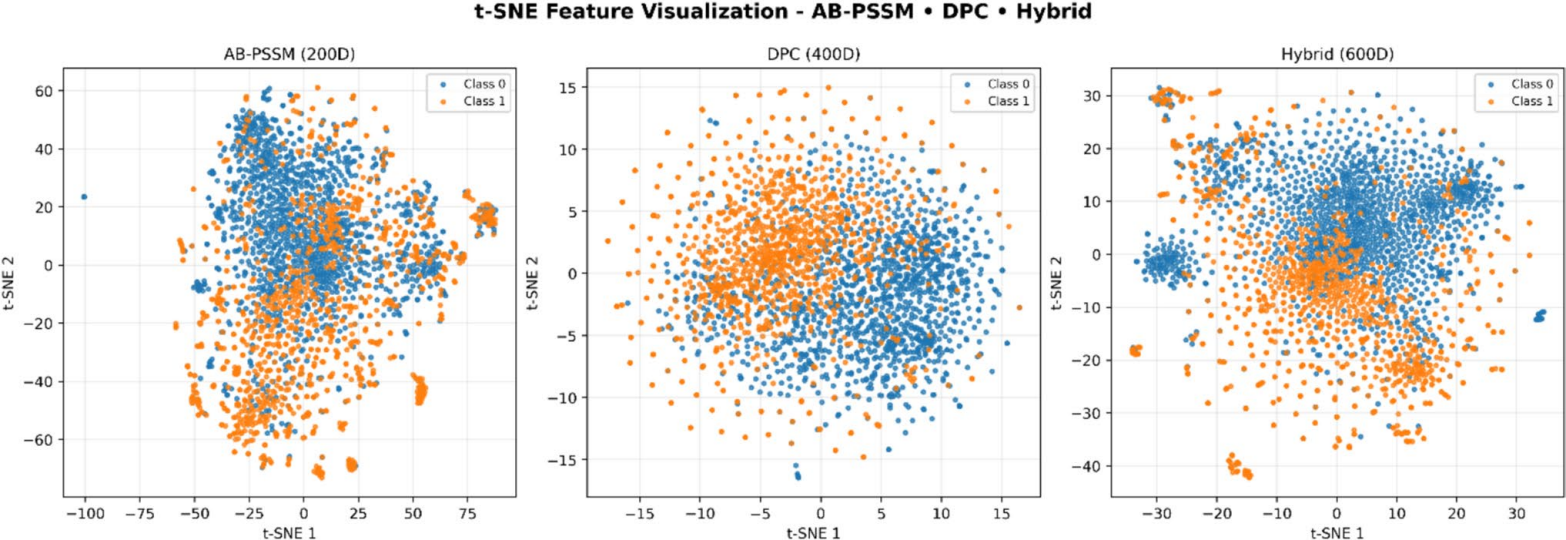
t-SNE visualization of feature using AB-PSSM, DPC, and Hybrid (600D)

The t-SNE projections Fig. 10 provided an orthogonal view on feature distinguishability. AB-PSSM and DPC independently generated largely overlapping clusters of druggable and non-druggable proteins, however the Hybrid representation resulted in more distinct groups, exhibiting a sharper delineation between the two categories. This observation aligns with the enhanced predictive efficacy of the Hybrid model and demonstrates that the integrated features provide superior discriminative structure compared to individual descriptors.

### Comparative discussion with existing methods

Several recent deep learning-based predictors for sequence-based protein and peptide prediction have been proposed, including DeepAIPs-SFLA, iSucc-SnCNs, pNPs-CapsNet, SBSM-Pro, DeepAIPs-Pred, and TargetAVP-DeepCaps. The DeepAIPs-SFLA model combines evolutionary and structural sequence characteristics with enhanced deep learning methodologies to improve peptide prediction efficacy [57]. iSucc-SnCNs utilizes hybrid deep neural architectures integrated with multi-view feature fusion to enhance resilience and generalization in protein modification prediction tasks [58]. The efficacy of capsule neural networks combined with protein language model embeddings for capturing hierarchical sequence relationships in biological sequence analysis is demonstrated by the recently proposed pNPs-CapsNet framework [59]. SBSM-Pro presents a scalable bio-sequence classification system with similarity kernel learning for protein prediction challenges [60]. Recent predictors such as DeepAIPs-Pred and TargetAVP-DeepCaps highlight the increasing utilization of sophisticated deep learning and capsule-based architectures for biological sequence modeling [61, 62]. Despite these promising developments, many existing approaches primarily emphasize predictive accuracy while providing limited statistical validation and interpretability analysis. This study enhances performance evaluation by out-of-fold benchmarking, statistical significance testing, and SHAP/t-SNE-based interpretation to ensure a more reliable and transparent assessment. A number of computational models have been developed to predict the existence of drugs that can bind to proteins, with most of them employing conventional machine learning and ensemble learning algorithms. The earlier methods, such as those proposed by Han et al. [24] and Jamali et al. [25], used support vector machine and hand-crafted sequence descriptors, and they usually had a prediction accuracy of between 80 and 85%. Newer ensemble-based predictors including DrugMiner and the stacked generalization frameworks introduced by Charoenkwan et al. [63] have shown average improvements with an overall accuracy score of around 87–88 percent. A model built by [64] is XGB-DrugPred, and XGBoost model that operated using optimized descriptors and achieved a performance of about 89, which is one of the strongest ensemble learning models to date.

The best competitive results have been reported in models combining deep learning or protein language models. Zhang et al. [65] employed ESM2-based embeddings combined with ensemble learning with an accuracy of about 90–91. The initial ESM framework was previously developed by Rives et al. (2021) [56], who showed that large protein language models can capture important biological signals in millions of sequences, with Bepler and Berger (2021) [53] later showing that these embeddings capture structural and functional relationships. In support of the growing importance of deep learning in protein biology, AlQuraishi (2019) [66] presented one of the first end-to-end differentiable protein structure prediction models. Although these methods have made an improvement over other older ensemble models, they have been evaluated based on traditional metrics and have not been statistically tested or interpretable at the feature level.

The present study presents better performance of discrimination than the past methods with ROC-AUC and PR-AUC scores consistently exceeding 95% and classification accuracy going up to 90%. The proposed methodology improves the raw predictive performance, adding the strict tests of statistical significance (DeLong, McNemar, and bootstrap analyses) and interpretation tools such as SHAP and t-SNE. Such combination is not only a way to prove that the observed improvements are statistically significant, but also to correlate the predicted outputs with the biologically significant features of a sequence. Therefore, the paper offers a better and more visible criterion of prediction of druggable proteins compared to the past deep learning or machine learning models (Table 8).

**Table 8:** Comparative performance of existing druggable protein prediction methods

Study / Year [Ref]	Features / representation	Model(s) used	Reported accuracy (%)	Notes
Han et al. 2007[24]	Basic sequence descriptors	SVM	~ 84	Early ML approach; limited feature space
Jamali et al. 2016 [25]	AAC, DPC, physicochemical	SVM, RF	80–85	Conventional sequence features
Charoenkwan et al. 2022 [63]	Multiple descriptors	Stacked ensemble	87–88	Best among ensemble ML at that time
Sikander et al. 2022[64]	Optimized handcrafted features	XGB (XGB-DrugPred)	~ 89	Strong ensemble baseline
Rives et al. 2021[56]	ESM embeddings (large-scale protein language model)	Unsupervised / downstream ML	–	Introduced ESM, foundation of protein language modeling
Bepler & Berger, 2021 [61]	Protein language embeddings	Deep embeddings	–	Showed embeddings capture structural/functional signals
AlQuraishi, 2019 [66]	Sequence-to-structure (end-to-end)	Deep learning	–	Early DL milestone in protein biology (structure-focused)
Borhani et al. 2025 [67]	ESM-2 embeddings + Gene Ontology	Deep learning (DrugTar)	AUC/PR-AUC ≈ 0.94	Recent SOTA embedding-based druggability model
Zhang et al. 2025[65][]	ESM2 embeddings	Ensemble learning	90–91	Latest deep embedding approach, no interpretability
Wang et al., 2024 [68]	Sequence embeddings, simulated features	GAN	85–88	Not benchmarked consistently
Ling et al.,2025[69]	Transformer embeddings	Transformer	85–88	Not benchmarked consistently
This study (2025)	Hybrid AB-PSSM + DPC (600D)	SVM, CapsBiLSTM, ResCapsNetPlus	≥ 90.5 (Acc), ≥ 95 (ROC/PR-AUC)	Adds rigorous stats (DeLong, McNemar, bootstrap) + SHAP/t-SNE interpretability

## Conclusion

The hybrid features developed by the combination of AB-PSSM and DPC representation managed to combine both evolutionary and compositional information leading to strong predictive performance. The SVM and CapsBiLSTM models achieved classification accuracies above 90% and ROC-AUC and PR-AUC scores above 95% establishing competitive performance on the benchmark dataset. The soundness of the proposed framework was tested with the help of different statistical tests, such as DeLong test, McNemar test, and bootstrap confidence intervals that showed that the difference in performance was statistically significant. Interpretability analyses with SHAP and t-SNE revealed that the predictions were biologically relevant sequence descriptors, which increased the transparency and confidence in the predictions. The comparative analysis shows that the framework is superior to the existing machine learning and ensemble-based frameworks and provides a benefit over the recent embedding-driven frameworks by combining accuracy, statistical rigor, and interpretability.

Although the promising results, many limitations of the suggested approach must be recognized. The proposed framework depends on evolutionary features that are obtained from PSSM profiles; nevertheless, the computationally challenging characteristics of these features may restrict their use in large-scale applications. The model was assessed on a singular benchmark dataset utilizing stratified five-fold out-of-fold validation, and validation on independent external datasets is required to further establish the model’s generalization capabilities. Moreover, while SHAP and t-SNE studies enhance interpretability, further biological confirmation of the revealed sequence patterns is essential. Future work will focus on improving the suggested model by combining protein language-model embeddings, investigating lightweight feature representations, and incorporating structural information when available to improve scalability and real-world application.

## Data Availability

No datasets were generated or analysed during the current study.
